# RvD1_n-3 DPA_ Downregulates the Transcription of Pro-Inflammatory Genes in Oral Epithelial Cells and Reverses Nuclear Translocation of Transcription Factor p65 after TNF-α Stimulation

**DOI:** 10.3390/ijms232314878

**Published:** 2022-11-28

**Authors:** Maria G. Balta, Olav Schreurs, Rashi Halder, Thomas M. Küntziger, Frank Sætre, Inger Johanne S. Blix, Espen S. Bækkevold, Enrico Glaab, Karl Schenck

**Affiliations:** 1 Institute of Oral Biology, University of Oslo, 0316 Oslo, Norway; 2 Luxembourg Centre for Systems Biomedicine (LCSB), University of Luxembourg, 4367 Belvaux, Luxembourg; 3 Department of Pathology, Oslo University Hospital, 0316 Oslo, Norway; 4 Institute of Clinical Dentistry, Faculty of Dentistry, University of Oslo, 0455 Oslo, Norway

**Keywords:** resolvin, specialized pro-resolving mediators, oral epithelium, gingival, oral inflammation, periodontitis, p65, NF-κB

## Abstract

Specialized pro-resolving mediators (SPMs) are multifunctional lipid mediators that participate in the resolution of inflammation. We have recently described that oral epithelial cells (OECs) express receptors of the SPM resolvin RvD1_n-3 DPA_ and that cultured OECs respond to RvD1_n-3 DPA_ addition by intracellular calcium release, nuclear receptor translocation and transcription of genes coding for antimicrobial peptides. The aim of the present study was to assess the functional outcome of RvD1_n-3 DPA_–signaling in OECs under inflammatory conditions. To this end, we performed transcriptomic analyses of TNF-α-stimulated cells that were subsequently treated with RvD1_n-3 DPA_ and found significant downregulation of pro-inflammatory nuclear factor kappa B (NF-κB) target genes. Further bioinformatics analyses showed that RvD1_n-3 DPA_ inhibited the expression of several genes involved in the NF-κB activation pathway. Confocal microscopy revealed that addition of RvD1_n-3 DPA_ to OECs reversed TNF-α-induced nuclear translocation of NF-κB p65. Co-treatment of the cells with the exportin 1 inhibitor leptomycin B indicated that RvD1_n-3 DPA_ increases nuclear export of p65. Taken together, our observations suggest that SPMs also have the potential to be used as a therapeutic aid when inflammation is established.

## 1. Introduction

The epithelial lining of the oral cavity represents a critical component of the host defense. Apart from shaping a passive physical barrier, oral epithelial cells (OECs) serve as a direct line of communication between the immune system and the external environment by generating and secreting cytokines, chemokines and other factors [1].

Acute inflammation aims to protect the host from microbial invasion or tissue injury. In a state of ‘immune fitness’, the inflammatory response remains contained in time and space, and is designed to resolve [2]. Although previously considered as a passive process, the resolution of acute inflammation is now recognized as an active host response, which is activated temporally after an acute challenge. Resolution is in part mediated by a range of specialized pro-resolving lipid mediators (SPMs), including lipoxins, resolvins, marezins and protectins [3]. Failure of the pro-resolving mechanisms prolongs pro-inflammatory activity, leading to chronic inflammation which may underlie the pathogenesis of chronic diseases [2,4]. In the oral cavity for example, the inability of the host to successfully control and resolve inflammation within the gingival tissues may contribute to the development of chronic periodontitis [4]. Therefore, understanding and controlling the activity of pro-resolving pathways may pave the way for the design of novel host-modulatory therapies.

Over the last few decades, SPMs have emerged as possible candidates for therapy and a number of studies have shown the anti-inflammatory and wound healing actions of SPMs on leukocytes, fibroblasts and bone cells [5,6,7,8,9,10]. Recently, their role on epithelial tissues has gained more attention [11,12]. Focus has primarily been on epidermal tissues, but we have previously described that the recently identified SPM, resolvin D1_n-3 DPA_ (RvD1_n-3 DPA_), can induce responses in primary OECs derived from oral mucosa, including intracellular calcium release, nuclear receptor translocation and transcription of genes coding for antimicrobial peptides [13].

Many cell cultural studies on SPMs investigated their role in dampening inflammation where the SPMs were added at the same time or before an inflammatory stimulus was introduced [14,15,16]. By contrast, in the present study, we aimed at testing the hypothesis that addition of RvD1_n-3 DPA_ to cell cultures of OECs after conditioning the cells with a pro-inflammatory stimulus (TNF-α addition) also could modulate the OECs’ response. First, we used mRNA sequencing (mRNAseq) to examine the effect on the gene expression profile of the differently treated cells and found a similar anti-inflammatory profile of RvD1_n-3 DPA_ as seen for other SPMs. Follow-up analysis of the RNA-seq results by bioinformatics and confocal microscopy showed that the anti-inflammatory effects of RvD1_n-3 DPA_ can be explained at least partly by reversal of the nuclear translocation of transcription factor p65.

## 2. Results

### 2.1. mRNA-seq Analysis

**Figure 1:**
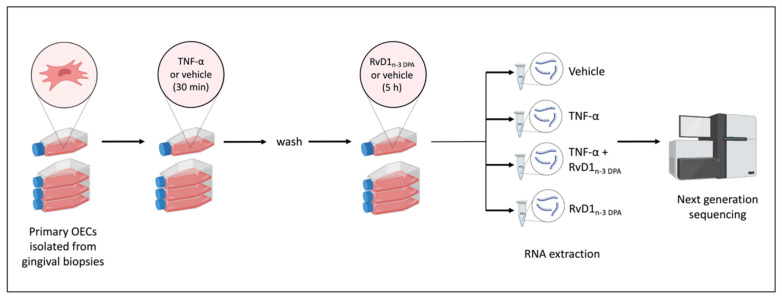
Experimental setup used for mRNA sequencing. Primary oral epithelial cells were incubated for 30 min with TNF-α or vehicle. After washing, RvD1_n-3 DPA_ or vehicle was added for 5 h. The cells were then harvested and processed for mRNA sequencing. Part of the figure was created with Biorender.com.

To investigate the effect of the newly identified SPM RvD1_n-3 DPA_ in an experimental setting associated with the presence of established inflammation, we analyzed by RNA-sequencing the gene expression profile of primary OECs without or with pre-stimulation with TNF-α and without or with addition of RvD1_n-3 DPA_ (Figure 1). The percentage of mapped reads varied between 97.1 and 98.7. Subsequent analysis identified 701 genes as differentially expressed (DEGs) between OECs treated with TNF-α versus vehicle (data not shown).

**Figure 2:**
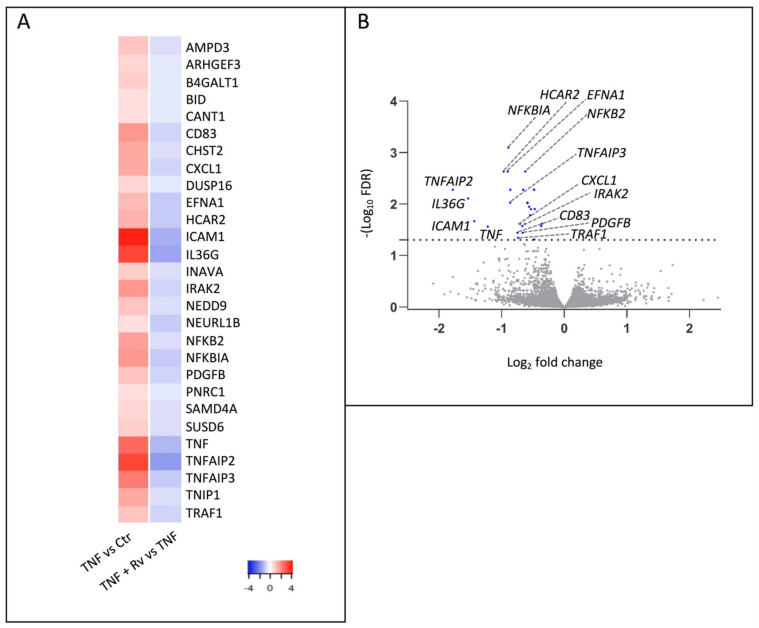
Differentially expressed genes in TNF-α-activated oral epithelial cells treated with RvD1_n-3 DPA_. (**A**) Based on FDR, 28 genes were differentially expressed between oral epithelial cells treated with TNF-α + RvD1_n-3 DPA_ versus TNF-α alone (blue). All genes were down-regulated. In red, the expression of the same genes is shown when TNF-α addition is compared with control (vehicle). (**B**) Volcano plot of the differentially expressed genes when TNF-α + RvD1_n-3 DPA_ and TNF-α addition were compared.

Furthermore, a total of 28 genes were filtered as differentially expressed genes (DEGs) between OECs treated with TNF-α + RvD1_n-3 DPA_ versus TNF-α (Figure 2A, B). All 28 DEGs found to be downregulated in cells treated with TNF-α + RvD1_n-3 DPA_ versus TNF-α alone (blue; Figure 2A) were transcripts that were significantly upregulated in primary OECs treated with TNF-α versus control (red; Figure 2A) and many of them with association to immune defenses.

### 2.2. GO Biological Process and KEGG Pathway Enrichment Analyses

**Figure 3:**
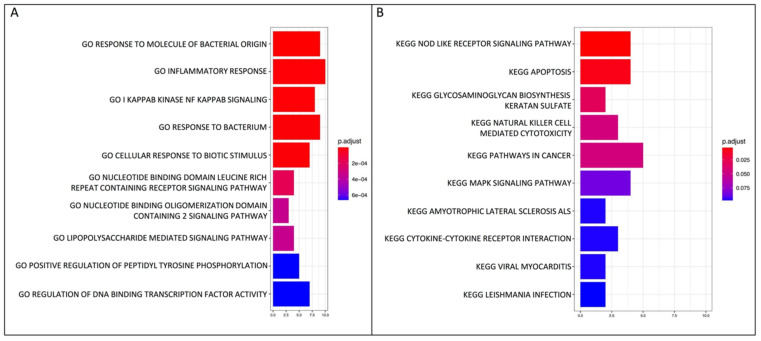
Gene ontology (GO) biological process and KEGG pathway enrichment analysis. (**A**) GO and (**B**) KEGG pathway enrichment analysis of biological processes on the 28 differentially expressed genes in oral epithelial cells when TNF-α + RvD1_n-3 DPA_ and TNF-α addition were compared.

Next, pathway enrichment analyses were performed using the Gene Ontology (GO) database and the KEGG database. The top 10 GO terms based on the 28 input DEGs are illustrated in Figure 3A and the top 50 GO terms are shown in Table S1. Ten genes were associated with inflammatory response (*NFKBIA, CHST2, IL36G, TNFAIP3, B4GALT1, ICAM1, CXCL1, TNIP1, IRAK2* and *TNF*), nine DEGs were associated with response to molecule of bacterial origin and to bacterium (*NFKBIA, NFKB2, IL36G, TNFAIP3, INAVA, ICAM1/CXCL1, IRAK2* and *TNF*), eight genes were associated with IkappaB kinase (IKK) signaling (*NFKBIA, IL36G, TNFAIP3, INAVA, CANT1, TNIP1, IRAK2* and *TNF*), and five and three genes were associated with positive and negative regulation of Nuclear Factor-kappa B (NF-*κ*B) signaling, respectively. Cellular response to lipid, intracellular receptor signaling pathway and negative regulation of DNA-binding transcription factor activity were also included among the significantly enriched biological processes (Table S1).

The KEGG pathway analysis highlighted that the “NOD-like receptor signaling pathway” and “apoptosis’’ were the top significantly enriched pathways among the 28 downregulated genes for TNF-α + RvD1_n-3 DPA_ versus TNF-α alone (Figure 3B).

**Figure 4:**
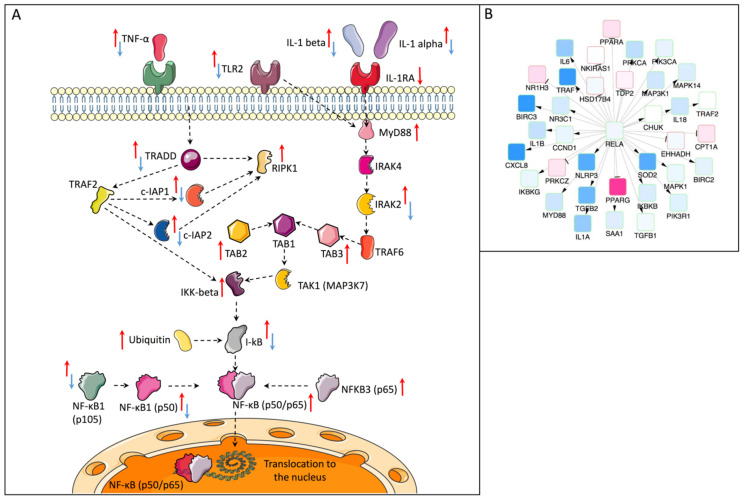
RvD1_n-3 DPA_ downregulates the NF-κB canonical pathway in TNF-α-activated oral epithelial cells. (**A**) The canonical pathway of NF-κB activation. Red arrows indicate transcriptional changes after TNF-α addition. Blue arrows show changes after addition of TNF-α + RvD1_n-3 DPA_. (**B**) Causal network analysis centered on *RELA*, showing that *RELA* regulates a variety of downstream pro-inflammatory targets for which RvD1_n-3 DPA_ appears to partly or fully reverse TNF-α-induced expression changes (based on TNF-α + RvD1_n-3 DPA_ and TNF-α comparison).

NF-κB signaling is crucial for the expression of a variety of genes amplifying inflammation, and this was among the statistically significant pathways that we found to be transcriptionally regulated by RvD1_n-3 DPA_. We therefore interrogated this pathway in more detail. Figure 4A summarizes the transcriptional changes within the canonical NF-κB signaling pathway in TNF-α-stimulated oral epithelial cells with or without subsequent incubation with RvD1_n-3 DPA_. TNF-α up-regulated 19 genes (red arrows) and down-regulated IL1RA and RvD1_n-3 DPA_ down-regulated 11 genes (blue arrows). RvD1_n-3 DPA_ counteracted transcription of many genes that were increased by TNF-α.

### 2.3. Causal Network Analysis (Causal Reasoning)

We used the entire human protein–protein interactions set with known direction and type of interaction (i.e., activating or inhibiting) from the STRING database for a causal network analysis [17] to identify key upstream regulatory genes/proteins, whose alterations can explain many of the downstream differential expression changes in their target genes/proteins.

After ranking according to significance level (Table S2), the top 50 key upstream genes identified included genes coding for 19 nuclear pore complex proteins (nucleoproteins), in addition to *TPR, NDC1, POM121* and *POM121C* which also code for structural constituents of the nuclear pore. *AAAS*, *RANBP2* and *RAE1*, coding for components that modulate the nuclear export and nucleocytoplasmic pathways were also among the 50 highest ranked genes. *XPO1* (coding for exportin 1), an important protein mediating nuclear export, was also highly ranked (nr. 59) and statistically significant in terms of expression changes controlled by this gene. This suggests that nucleocytoplasmic shuttling in the OECs is modulated after incubation of TNF-α-stimulated cells with RvD1_n−3 DPA_ as compared to TNF-α alone.

Using the protein–protein interactions from STRING, we also created a network visualization for *RELA*, the gene coding for the transcription factor p65, a major functional component of the NF-κB complex. The causal reasoning analysis identified *RELA* among the significant candidate regulatory genes indicating the potential role of p65 in regulating the genes for which RvD1_n-3 DPA_ appears to partly or fully reverse TNF-α-induced changes. Figure 4B shows the network of *RELA* transcriptional target changes whose expression in the TNF-α + RvD1_n-3 DPA_ versus TNF-α comparison changed in a manner that is consistent with the decreased expression of *RELA* and the regulation type (activating or inhibiting). This indicates that regulation by transcription factor p65 is a key component in the induction of responses seen in the OECs after incubation of TNF-α-stimulated cells with RvD1_n-3 DPA_ as compared to TNF-α alone.

### 2.4. Nuclear Localization of p65

**Figure 5:**
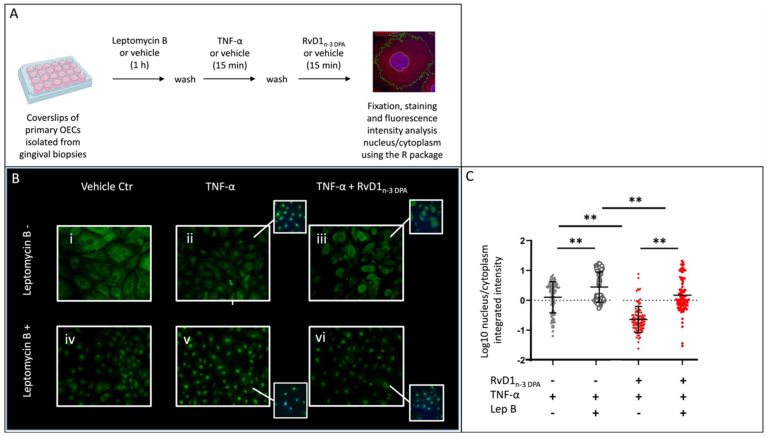
RvD1_n-3 DPA_ inhibits p65 translocation to the cell nucleus. (**A**) Experimental setup. Primary oral epithelial cells were treated for 1 h with leptomycin B or left untreated. Then, the cells were washed and incubated for 15 min with TNF-α or left untreated. After a final washing, RvD1_n-3 DPA_ or vehicle was added for 15 min after which the cover slips with the cells were processed for immunocytofluorescence and analysis with CellProfiler (see Material and Methods). (**B**) Microscopic pictures of p65 immunocytofluorescence (green) of cells as described in (**A**). Stainings combined with DAPI are displayed in Figure S1. Original magnification ×20. (**C**) Distribution of p65 in oral epithelial cells treated as indicated on the *x*-axis. The *y*-axis shows the ratio of nuclear/cytoplasmic p65 staining. Part of Figure 5A was created with BioRender.com. ** Statistically significant differences (*p* < 0.05) as assayed by repeated measures ANOVA with adjustment for multiple comparisons based on False Discovery Rate (FDR).

The causal network analyses above indicated that (1) nucleocytoplasmic shuttling in the OECs is modulated after incubation of TNF-α-stimulated cells with RvD1_n-3 DPA_ as compared to TNF-α alone, and that (2) regulation by transcription factor p65 can be a key component in the induction of responses seen in the OECs after this treatment. We therefore examined the effect of RvD1_n-3 DPA_ on the localization of p65 within the OECs after TNF-α stimulation (Figure 5A). In untreated OECs, p65 was mainly localized in the cytoplasm (Figure 5Bi). In cells treated with TNF-α, p65 translocated to the nucleus (Figure 5Bii,C). However, when the cells were subsequently treated with RvD1_n-3 DPA_, nuclear translocation was abolished and p65 accumulated in the perinuclear region (Figure 5Biii, C). We then compared these observations with experiments using the same conditions, but where OECs were first treated with leptomycin B to inhibit nuclear export via inhibition of chromosomal region maintenance (CRM1)/exportin 1 (XPO1). Exportin 1 is required for nuclear export of proteins containing a nuclear export sequence (NES). Leptomycin B addition led to increased p65 nuclear staining compared to untreated cells as expected, but cytoplasmic staining remained visible (Figure 5Biv,C). Exposure to TNF-α increased p65 nuclear translocation (Figure 5Bv,C) but leptomycin B treatment inhibited the nuclear efflux that was seen after RvD1_n-3 DPA_ addition (compare Figure 5Biii,vi,C). This shows that RvD1_n-3 DPA_ is no longer able to reverse nuclear translocation of p65 when CRM1/XPO1-mediated nuclear export is impaired.

## 3. Discussion

Specialized pro-resolving mediators (SPMs) can exert anti-inflammatory and wound healing-promoting actions on leukocytes, fibroblasts and bone cells [5,6,7,8,9,10]. Specifically, the recently characterized resolvin RvD1_n-3 DPA_ exhibits potent anti-inflammatory effects on human neutrophils and endothelial cells and has been shown to be produced by polymorphonuclear granulocytes and monocyte/macrophages [18,19,20]. To date, two G-protein coupled receptors for RvD1_n-3 DPA_ have been described: the formyl peptide receptor 2 (FPR2/ALX) and the G protein-coupled receptor 32 (DRV1/GPR32) [19]. We have recently described that oral epithelial cells (OECs) express these receptors and that cultured OECs respond to RvD1_n-3 DPA_ addition by intracellular calcium release, receptor translocation to the nucleus and transcription of genes coding for antimicrobial peptides [13].

Here, we wanted to explore the broader response of TNF-α-stimulated OECs to RvD1_n-3 DPA_. To this end, we carried out mRNA sequencing of cultured TNF-α-activated OECs, subsequently exposed to RvD1_n-3 DPA_ or to vehicle. In many studies, SPMs are added before or together with an inflammatory stimulus [6,14,21,22,23]. In contrast, our model consisted of first stimulating the cultured cells with TNF-α, after which RvD1_n-3 DPA_ was added (Figure 1). This means that the cells were brought into an inflammation-resembling state at the moment when they were exposed to RvD1_n-3 DPA_ and that our model therefore can indicate whether RvD1_n-3 DPA_ can reverse an inflammatory response, not only prevent it.

Based on mRNA sequencing, Gene Ontology and KEGG pathway analyses of the transcription data, comparing OECs treated with TNF-α + RvD1_n-3 DPA_ versus TNF-α alone showed that RvD1_n-3 DPA_ addition affected immune and inflammatory responses, including NF-κB signaling, NOD signaling, lipopolysaccharide signaling, MAPK signaling, natural killer cell mediated cytotoxicity and apoptosis. Such responses have also been described for other SPMs. For example, RvD3 and aspirin-triggered RvD3 downregulate the expression of the NF-κB protein and induce the expression of the NF-κB inhibitor protein in lung epithelial cells upon binding to ALX/FPR2 receptor [24]. Aspirin-triggered RvD3 also inhibits TNF-α–induced NF-κB activation in an in vivo model of acute lung injury [15]. Similarly, RvE1 treatment blocks the activation of the mitogen-activated protein kinase (MAPK) and nuclear factor (NF)-κB signaling pathways, and this inhibition contributes to the improvement in the inflammatory response induced by lipopolysaccharide (LPS) in the myocardial tissue of mice with LPS-induced myocardial injury [16]. In peritoneal macrophages, RvD1 and RvD2 attenuate the activation of nucleotide-binding domain leucine-rich repeat-containing protein 3 (NLRP3) inflammasome induced by LPS and palmitate [25]. Taken together, this indicates that RvD1_n-3 DPA_ has effects that also have been described for other SPMs.

To identify genes and processes that were affected by RvD1_n-3 DPA_ treatment the network causal reasoning analysis of known and predicted protein–protein interactions from the STRING database was then applied to the mRNA-seq data. After ranking according to significance level, more than half of the top 50 genes included genes that code for proteins involved in nucleocytoplasmic transport. Therefore, we decided to examine this process in the context of translocation of the transcription factor NF-κB p65 in response to TNF-α. NF-kB p65 is one of the main functional components of the NF-κB signaling pathway [26] which additionally—based on our mRNAseq data—was found to be affected by RvD1_n-3 DPA_ treatment. Causal reasoning analysis confirmed that *RELA* (coding for p65) is a significant candidate gene that could regulate the genes which RvD1_n-3 DPA_ appeared to affect after TNF-α challenge.

The complex between p65 and p50 is the most common heterodimer among the NF-κB dimers and is the functional component participating in nuclear translocation and activity of NF-κB. The p65/p50 complex translocates to the nucleus where it binds to response elements on the DNA [26]. In our setup, OECs were first treated for 15 min with TNF-α. This resulted in nuclear translocation of p65. When the cells thereafter were incubated with RvD1_n-3 DPA_ for 15 min, the p65 was shuttled back to the cytoplasm. This was not seen when RvD1_n-3 DPA_ was substituted by vehicle. The ability to reside in the nucleus is essential for transcription factor (TF) activity and cytoplasmic TF re-localization can serve as an inactivation mechanism. This suggests that RvD1_n-3 DPA_, by reversing the TNF-α–induced translocation of p65, can attenuate NF-κB activity.

To probe into the mechanism of the nuclear translocation reversal of p65 by RvD1_n-3 DPA_, we used leptomycin B, a known irreversible inhibitor of nuclear export protein exportin 1 (coded by *XPO1/CRM1*) [27]. When OECs were pre-incubated with leptomycin B, nuclear translocation was still seen after TNF-α addition, but it was not reversed when RvD1_n-3 DPA_ was added subsequently. This strongly suggests that the effect of RvD1_n-3 DPA_ on the reversed nuclear localization of p65 is achieved through increased nuclear export of p65.

Diseases driven by chronic inflammation, e.g., chronic infections such as periodontitis, allergies and autoimmune diseases, need improved treatment options such as modulation of chronic inflammation without causing immunosuppression. Over the last few decades, SPMs have emerged as promising therapeutic alternatives because they can provide anti-inflammatory and pro-resolving actions without being immunosuppressive [10]. In this respect, RvD1_n-3 DPA_ can be a promising candidate.

The present inductive study is an in vitro assessment of primary OECs, first exposed to a pro-inflammatory and then to an anti-inflammatory stimulus. The complex dynamic milieu shaped upon microbial–host interactions and the possible modifying complexity of various inflammatory stimuli in vivo is lacking. In vivo studies in animals and then humans are therefore required to validate the effect of RvD1_n-3 DPA_ on chronic inflammation. Experimentally induced (in animals) or natural (in humans) forms of periodontitis can constitute valid study models [23]. Further studies will also address in more detail the mechanisms behind the reversal by RvD1_n-3 DPA_ of NF-κB nuclear translocation in OECs and potentially also in other cell types.

Taken together, RvD1_n-3 DPA_ acts as an SPM on oral epithelial cells. When applied after TNF-α stimulation, RvD1_n-3 DPA_ reverses nuclear translocation of the transcription factor p65. This indicates that RvD1_n-3 DPA_ has the potential to be used as a therapeutic aid after inflammation is established, not only as a preventive measure.

## 4. Material and Methods

### 4.1. Biopsy Material

Biopsies for cell culture were obtained from the healthy buccal gingiva in volunteers undergoing tooth extractions (*n* = 4, mean age ± SD = 35.3 ± 12 years, 2 females and 2 males). Probing depth at the biopsy sites was <5 mm, clinical attachment loss was ≤2 mm, and there was no bleeding on probing. The study was conducted according to the guidelines of the Declaration of Helsinki, and approved by the Regional Committee for Medical Research Ethics in South-East Norway (nr. 2017/2196). Informed consent was obtained from all subjects involved in the study.

### 4.2. Isolation of Primary Oral Epithelial Cells

OECs were isolated from biopsies as described previously [28]. Briefly, biopsies were transferred to Dulbecco’s modified Eagle medium with 1.25 mg/mL dispase (GIBCO, Thermo Fisher Scientific, Waltham, MA, USA) and incubated over night at 4 °C. The epithelial sheets were peeled off, cut into small pieces and incubated in 10X trypsin EDTA (Sigma-Aldrich, St Louis, MO, USA) for 7 min at 37 °C. A Pasteur pipette was used to loosen the cells and enzymatic treatment was stopped by the addition of fetal calf serum (FCS). The cells were then cultured in keratinocyte serum-free medium (KSFM, GIBCO), supplemented with 25 μg/mL bovine pituitary extract (BPE; GIBCO), 1 ng/mL epidermal growth factor (EGF) and 1% Antibiotic-Antimycotic (GIBCO), in a humidified atmosphere of 5% CO_2_ in air at 37 °C. For all experiments, the cells were seeded at a density of 500,000 cells per well in 6-well plates, incubated overnight and then grown in KSFM without addition of BPE and EGF 24 h before stimulation. All the cells used in the experiments were between passage 3 and passage 6 and they were 80–90% confluent at the time of the experiment.

### 4.3. Resolvin

RvD1_n-3 DPA_ was prepared by total organic synthesis [19]. The structural integrity of RvD1_n-3 DPA_ was monitored using UV tandem LC-MS/MS and matched against authentic material of RvD1_n-3 DPA_. Before use, RvD1_n-3 DPA_, diluted in pure ethanol, was resuspended in phosphate-buffered saline to a dilution of 1:100.

### 4.4. RNA High-Throughput Sequencing and Data Processing

Primary OECs were isolated from gingival biopsies and cultured in KSFM (5% CO_2_, 37 °C). Cells seeded at a density of 2 × 10^6^ cells in T25 flasks were stimulated with TNF-α or vehicle (KSFM, containing <0.1% ethanol) for 30 min (5% CO_2_, 37 °C), washed twice with PBS and then incubated with RvD1_n-3 DPA_ (0.1 nM) or vehicle (KSFM, containing <0.1% ethanol) for 5 h (5% CO_2_, 37 °C) (Figure 1). Cells were washed twice with PBS before lysis in RLT buffer (Qiagen, Kista, Sweden) supplemented with 1% β-mercaptoethanol (Sigma-Aldrich). Total RNA was extracted using the QIAcube and the QIAcube standard RNeasy mini kit (Qiagen) using the DNase digestion protocol, RNA libraries were generated and paired-end sequencing was performed using a read length of 150 bp. The experimental profiling analyses were followed by computational data quality control, pre-processing and analysis, as outlined below.

The raw RNA-seq fastq files were quality-checked using the FastQC software [29], and the data were pre-processed using the software package Rsubread (version 1.32.2, Developers: Wei Shi et al., University of Melbourne, Parkville, Victoria, Australia) [30]. Gene-level differential expression analysis was conducted in the R statistical programming software (version 3.5.1, Developers: R Core Team, Vienna, Austria) [31] using the software package edgeR [32], and filtering out genes with low expression counts using the filter ByExpr-function with default parameters. Normalization factors to scale the raw library size were determined using the calcNormFactors-function with default settings, and posterior dispersion estimates were obtained by applying the estimateDisp-function with the robust-parameter set to true in order to robustify the estimation against outliers.

Pathway enrichment analyses were implemented using the R software package clusterProfiler [33] and gene set collections for the KEGG and Gene Ontology databases obtained from the MSigDB repository [34]. As input for the enrichment analyses using Fisher’s Exact test, the gene-level differential expression analysis results obtained with edgeR workflow were used, filtering the significant differential genes to include only those with a false-discovery rate (FDR) below 0.05. Similarly, *p*-value significance scores for the pathway over-representation analysis were also adjusted to obtain final FDR scores according to the method by Benjamini and Hochberg [35].

Next, a causal network analysis (causal reasoning analysis) [17] was also applied to the differentially expressed genes derived from the edgeR analysis, using direct activating and inhibiting human protein–protein interactions obtained from the Search Tool for the Retrieval of Interacting Genes/Proteins (STRING) [36]. STRING is a biological database that collects, scores and integrates publicly available sources of protein–protein interaction information and complements these with computational predictions of potential associations. This resource was used to assemble a comprehensive and objective global protein interaction network, covering direct (physical) interactions in humans. Specifically, the current full collection of human activating or inhibiting protein–protein interactions was downloaded from the STRING database (v11.5) and provided as input to the network causal reasoning analysis, together with the differentially expressed genes. The causal reasoning analysis identifies key upstream regulatory genes/proteins, whose alterations and known activating and inhibiting interactions can explain many of the downstream differential expression changes in their direct target genes [17].

The data discussed in this publication have been deposited in NCBI’s Gene Expression Omnibus and are accessible through GEO Series accession.

### 4.5. Immunocytofluorescence and Fluorescence Intensity Analysis

Primary OECs were seeded on glass coverslips at a density of 250,000 cells per well in a 24-well culture plate. For the nuclear export experiments, half of the coverslips were incubated with leptomycin B (20 ng/mL; Sigma-Aldrich) for 1 h and washed twice with PBS. The other half were incubated with vehicle and used as negative controls. Then, cells were exposed to TNF-α (final concentration 50 ng/mL; PeproTech, Thermo Fisher Scientific, Waltham, MA, USA) or vehicle (KSFM) for 15 min. The medium was removed, and the cells were then incubated with KSFM containing RvD1_n-3 DPA_ (0.1 nM) or vehicle (KSFM, containing <0.1% ethanol) for 15 min. The incubations were performed in a humidified atmosphere of 5% CO_2_ in air at 37 °C. The cells were washed briefly with PBS and fixed in 4% formaldehyde for 10 min at room temperature. After washing, the cells were stained and examined as described below.

Coverslips were treated with 0.1% Triton-X100 in 0.1% sodium citrate (*w*/*v*) before staining to permeabilize the attached cells. Then, coverslips were immersed in 5% normal horse serum and incubated overnight with 1 μg/mL unlabeled mouse anti-p65 at 4 °C (Santa Cruz Biotechnology, Dallas, TX, USA). After washing, coverslips were incubated with biotinylated horse anti-mouse IgG (Vectorlabs, Kirtlington, UK) and with Cy2-conjugated streptavidin (GE LifeSciences, Ctiva, Marlborough, MA, USA). Nuclei were stained with 4′,6-diamidino-2-phenylindole (DAPI, ThermoFisher Scientific). Images were taken using using a SuperApochromat 603/1.35 oil objective on an Olympus Fluoview FV1000 laser scanning confocal microscope and image overlays mounted using Adobe Photoshop CS5.

Mean fluorescence intensity in the nucleus versus intensity in the cytoplasm of primary OECs treated as mentioned above was analyzed using the CellProfiler v 3.1.0 (https://cellprofiler.org/about, accessed on September 2020) and R-software and graphs were constructed using GraphPad Prism v8.0. (GraphPad Software) (Figure S1).

## Data Availability

The data described in this publication have been deposited in NCBI’s Gene Expression Omnibus and are accessible through GEO Series accession, number GSE215275.

## Supplementary Materials

The supporting information can be downloaded at: https://www.mdpi.com/article/10.3390/ijms232314878/s1.
