# Supplementary material for: RvD1n-3 DPA Downregulates the Transcription of Pro-Inflammatory Genes in Oral Epithelial Cells and Reverses Nuclear Translocation of Transcription Factor p65 after TNF-α Stimulation
Source: Int J Mol Sci. 2022 Nov 28;23(23):14878. doi: 10.3390/ijms232314878 (PMC9737907; doi:10.3390/ijms232314878)
Supplement: Supplementary file 1 [file ijms-23-14878-s001.zip › Figure S1.pdf]

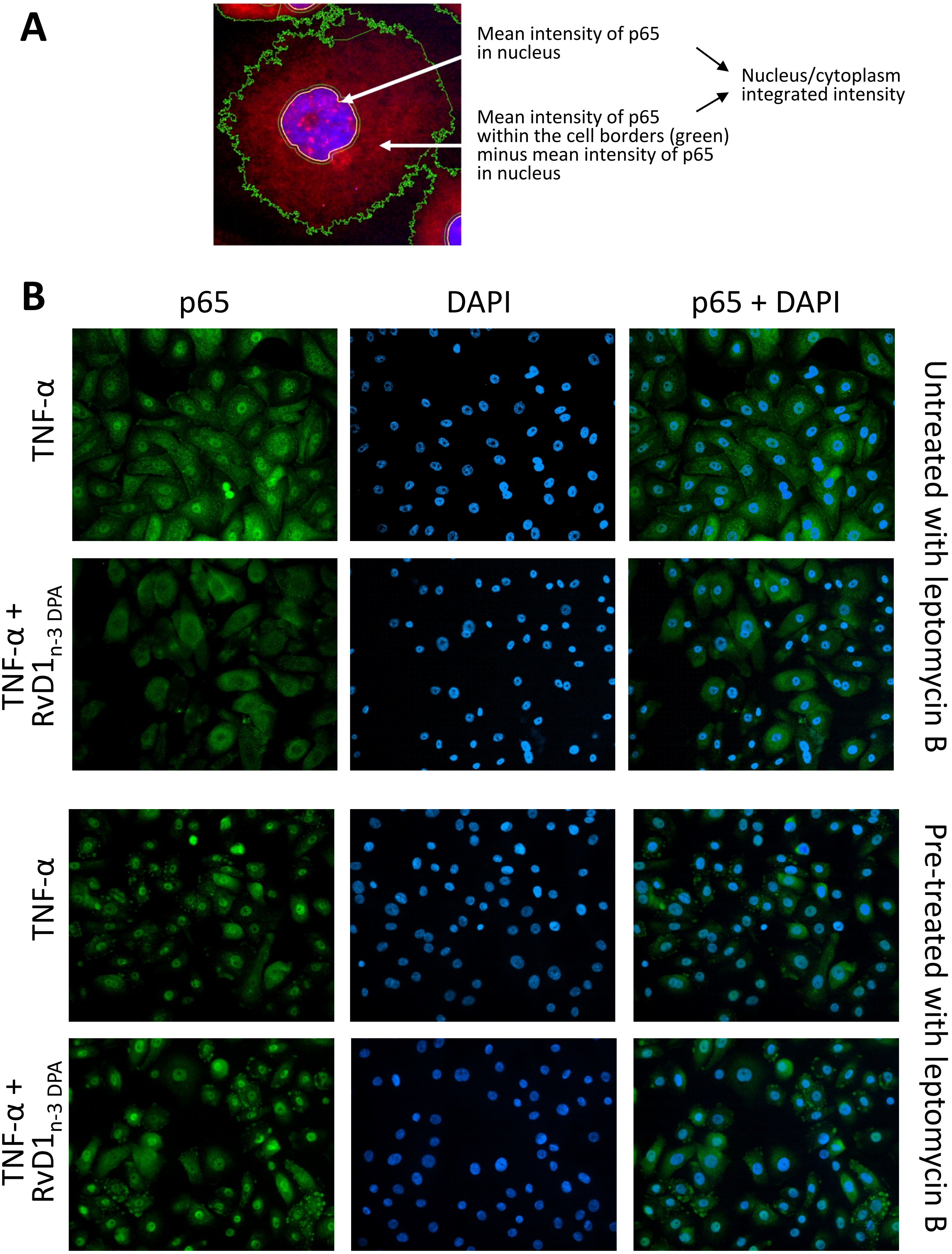

Figure S1. CellProfiler analysis of mean fluorescence intensity of p65 in the nucleus versus the cytoplasm of primary OECs. A. The nucleus/cytoplasm integrated intensity was calculated using the CellProfiler v 3.0 and the R-software. **B.** Examples of the p65 stainings used in Figure 5, including staining for cell nuclei (DAPI). Upper and lower panels without and with pre-treatment with leptomycin B, respectively (indicated at the right). Cells were stimulated with TNF-α or with TNF-α and $RvD1_{n\text{-}3\ DPA}$ (indicated at the left).
