# Supplementary material for: RvD1n-3 DPA Downregulates the Transcription of Pro-Inflammatory Genes in Oral Epithelial Cells and Reverses Nuclear Translocation of Transcription Factor p65 after TNF-α Stimulation
Source: Int J Mol Sci. 2022 Nov 28;23(23):14878. doi: 10.3390/ijms232314878 (PMC9737907; doi:10.3390/ijms232314878)
Supplement: Supplementary file 1 [file ijms-23-14878-s001.zip › Table S1.pdf]

Table S1. Top 50 GO terms in the biological process enrichment analysis using the GO database

ID	gene ID	p value *	q value **
GO RESPONSE TO MOLECULE OF BACTERIAL ORIGIN	NFKBIA/NFKB2/IL36G/TNFAIP3/INAVA/ICAM1/CXCL1/IRAK2/TNF	7.65566E-10	6.56775E-07
GO INFLAMMATORY RESPONSE	NFKBIA/CHST2/IL36G/TNFAIP3/B4GALT1/ICAM1/CXCL1/TNIP1/IRAK2/TNF	8.52624E-09	3.65731E-06
GO I KAPPAB KINASE NF KAPPAB SIGNALING	NFKBIA/IL36G/TNFAIP3/INAVA/CANT1/TNIP1/IRAK2/TNF	1.57637E-08	4.50787E-06
GO RESPONSE TO BACTERIUM	NFKBIA/NFKB2/IL36G/TNFAIP3/INAVA/ICAM1/CXCL1/IRAK2/TNF	3.69371E-08	6.56743E-06
GO CELLULAR RESPONSE TO BIOTIC STIMULUS	NFKBIA/IL36G/TNFAIP3/ICAM1/CXCL1/IRAK2/TNF	3.82764E-08	6.56743E-06
GO NUCLEOTIDE BINDING DOMAIN LEUCINE RICH REPEAT CONTAINING RECEPTOR SIGNALING PATHWAY	NFKBIA/TNFAIP3/INAVA/IRAK2	8.39255E-07	0.000119999
GO NUCLEOTIDE BINDING OLIGOMERIZATION DOMAIN CONTAINING 2 SIGNALING PATHWAY	NFKBIA/TNFAIP3/INAVA	1.91044E-06	0.000230283
GO LIPOPOLYSACCHARIDE MEDIATED SIGNALING PATHWAY	NFKBIA/TNFAIP3/IRAK2/TNF	2.14742E-06	0.000230283
GO POSITIVE REGULATION OF PEPTIDYL TYROSINE PHOSPHORYLATION	EFNA1/NEDD9/ICAM1/TNF/PDGFB	5.14377E-06	0.00044416
GO REGULATION OF DNA BINDING TRANSCRIPTION FACTOR ACTIVITY	NFKBIA/NFKB2/TNFAIP3/ICAM1/IRAK2/TNF/TRAF1	5.17732E-06	0.00044416
GO REGULATION OF EXTRINSIC APOPTOTIC SIGNALING PATHWAY	TNFAIP3/ICAM1/BID/TNF/TRAF1	6.27324E-06	0.000489253
GO CYTOPLASMIC PATTERN RECOGNITION RECEPTOR SIGNALING PATHWAY	NFKBIA/TNFAIP3/INAVA/IRAK2	7.04326E-06	0.000500743
GO POSITIVE REGULATION OF MAPK CASCADE	EFNA1/IL36G/INAVA/ICAM1/IRAK2/TNF/PDGFB	8.13439E-06	0.000500743
GO POSITIVE REGULATION OF NF KAPPAB TRANSCRIPTION FACTOR ACTIVITY	NFKB2/ICAM1/IRAK2/TNF/TRAF1	8.17164E-06	0.000500743
GO RESPONSE TO MURAMYL DIPEPTIDE	NFKBIA/TNFAIP3/INAVA	9.78691E-06	0.000559742
GO EPITHELIAL CELL APOPTOTIC PROCESS	TNFAIP3/ICAM1/BID/TNF	1.31842E-05	0.000706914
GO PATTERN RECOGNITION RECEPTOR SIGNALING PATHWAY	NFKBIA/TNFAIP3/INAVA/TNIP1/IRAK2	1.42037E-05	0.00071678
GO EXTRINSIC APOPTOTIC SIGNALING PATHWAY VIA DEATH DOMAIN RECEPTORS	TNFAIP3/ICAM1/BID/TNF	1.56209E-05	0.000729194
GO CYTOKINE RECEPTOR BINDING	IL36G/CXCL1/BID/TNF/TRAF1	1.61496E-05	0.000729194
GO LEUKOCYTE MIGRATION	IL36G/B4GALT1/ICAM1/CXCL1/TNF/PDGFB	1.78774E-05	0.000766845
GO CELLULAR RESPONSE TO LIPID	NFKBIA/IL36G/TNFAIP3/ICAM1/CXCL1/IRAK2/TNF	2.61005E-05	0.001066262
GO REGULATION OF PEPTIDYL TYROSINE PHOSPHORYLATION	EFNA1/NEDD9/ICAM1/TNF/PDGFB	3.17269E-05	0.001237198
GO INTERLEUKIN 6 PRODUCTION	IL36G/TNFAIP3/INAVA/TNF	4.12021E-05	0.001536828
GO EXTRINSIC APOPTOTIC SIGNALING PATHWAY	TNFAIP3/ICAM1/BID/TNF/TRAF1	4.35135E-05	0.001555418
GO ENDOTHELIAL CELL APOPTOTIC PROCESS	TNFAIP3/ICAM1/TNF	5.03544E-05	0.001727952
GO POSITIVE REGULATION OF PROTEIN TYROSINE KINASE ACTIVITY	EFNA1/NEDD9/PDGFB	7.05829E-05	0.002242692
GO TUMOR NECROSIS FACTOR RECEPTOR SUPERFAMILY BINDING	BID/TNF/TRAF1	7.05829E-05	0.002242692
GO TOLL LIKE RECEPTOR SIGNALING PATHWAY	NFKBIA/TNFAIP3/TNIP1/IRAK2	7.71952E-05	0.002253692
GO POSITIVE REGULATION OF DEFENSE RESPONSE	NFKBIA/TNFAIP3/INAVA/TNIP1/IRAK2/TNF	7.8564E-05	0.002253692
GO POSITIVE REGULATION OF DNA BINDING TRANSCRIPTION FACTOR ACTIVITY	NFKB2/ICAM1/IRAK2/TNF/TRAF1	7.88101E-05	0.002253692

GO RESPONSE TO TUMOR NECROSIS FACTOR	NFKBIA/TNFAIP3/ICAM1/TNF/TRAF1	0.000102108	0.002825753
GO ACTIVATION OF INNATE IMMUNE RESPONSE	NFKBIA/TNFAIP3/INAVA/TNIP1/IRAK2	0.000135635	0.003636267
GO REGULATION OF EPITHELIAL CELL APOPTOTIC PROCESS	TNFAIP3/ICAM1/TNF	0.000171033	0.004315531
GO REGULATION OF TUMOR NECROSIS FACTOR MEDIATED SIGNALING PATHWAY	TNFAIP3/TNF/TRAF1	0.000171033	0.004315531
GO PEPTIDYL TYROSINE MODIFICATION	EFNA1/NEDD9/ICAM1/TNF/PDGFB	0.000177047	0.004339637
GO TUMOR NECROSIS FACTOR MEDIATED SIGNALING PATHWAY	NFKBIA/TNFAIP3/TNF/TRAF1	0.000188504	0.004492116
GO POSITIVE REGULATION OF LEUKOCYTE ADHESION TO VASCULAR ENDOTHELIAL CELL	ICAM1/TNF	0.000197211	0.004572603
GO REGULATION OF PROTEIN SERINE THREONINE KINASE ACTIVITY	DUSP16/TNFAIP3/INAVA/IRAK2/TNF/PDGFB	0.000205885	0.004648095
GO REGULATION OF RESPONSE TO CYTOKINE STIMULUS	TNFAIP3/IRAK2/TNF/TRAF1	0.000216547	0.00476346
GO REGULATION OF MAP KINASE ACTIVITY	DUSP16/INAVA/IRAK2/TNF/PDGFB	0.000235552	0.004916898
GO ACUTE INFLAMMATORY RESPONSE	B4GALT1/ICAM1/TNF	0.000238257	0.004916898
GO CHRONIC INFLAMMATORY RESPONSE	TNFAIP3/TNF	0.000240717	0.004916898
GO POSITIVE REGULATION OF KINASE ACTIVITY	EFNA1/NEDD9/INAVA/IRAK2/TNF/PDGFB	0.000249464	0.00497706
GO NEGATIVE REGULATION OF LIPID CATABOLIC PROCESS	HCAR2/TNF	0.000288478	0.005624634
GO EXTERNAL SIDE OF PLASMA MEMBRANE	B4GALT1/ICAM1/TNF/CD83	0.000303446	0.005677232
GO POSITIVE REGULATION OF INTERLEUKIN 6 PRODUCTION	IL36G/INAVA/TNF	0.000305767	0.005677232
GO EPITHELIAL CELL DEVELOPMENT	B4GALT1/ICAM1/TNF/PDGFB	0.000311029	0.005677232
GO NEGATIVE REGULATION OF NF KAPPAB TRANSCRIPTION FACTOR ACTIVITY	NFKBIA/TNFAIP3/IRAK2	0.000320598	0.005729978
GO ANGIOGENESIS INVOLVED IN WOUND HEALING	TNFAIP3/B4GALT1	0.000340478	0.00579071
GO GERMINAL CENTER FORMATION	NFKB2/TNFAIP3	0.000340478	0.00579071

*p-value indicates nominal significance, **q-value indicates significance after adjustments for multiple hypothesis testing.
