# Supplementary material for: RvD1n-3 DPA Downregulates the Transcription of Pro-Inflammatory Genes in Oral Epithelial Cells and Reverses Nuclear Translocation of Transcription Factor p65 after TNF-α Stimulation
Source: Int J Mol Sci. 2022 Nov 28;23(23):14878. doi: 10.3390/ijms232314878 (PMC9737907; doi:10.3390/ijms232314878)
Supplement: Supplementary file 1 [file ijms-23-14878-s001.zip › Table S2.pdf]

Table S2. Top 50 upstream genes identified in the causal network analysis using the STRING database.

Gene Symbol	Rank	p value *	Enrichment **
SKIV2L2	1	3.82E-11	1.41E-08
EXOSC10	2	8.69E-11	2.36E-09
**NUP107**	2	4.84E-07	1.80E-15
**NUP85**	2	4.84E-07	1.80E-15
**NUP133**	2	4.84E-07	1.80E-15
**NUP43**	2	4.84E-07	1.80E-15
**NUP160**	2	4.84E-07	1.80E-15
**NUP37**	2	4.84E-07	1.80E-15
**RANBP2**	9	2.31E-06	9.38E-17
SRSF10	10	6.80E-10	7.13E-11
RPS27	11	3.10E-05	4.91E-10
DIS3	12	1.43E-09	6.35E-07
**AAAS**	12	1.27E-06	5.09E-10
**NUP155**	12	1.27E-06	5.09E-10
**NUPL2**	12	1.27E-06	5.09E-10
**NUP54**	12	1.27E-06	5.09E-10
**NUP205**	12	1.27E-06	5.09E-10
**NUP35**	12	1.27E-06	5.09E-10
**NUP93**	12	1.27E-06	5.09E-10
**NUP50**	12	1.27E-06	5.09E-10
**TPR**	12	1.27E-06	5.09E-10
**NDC1**	12	1.27E-06	5.09E-10
**NUP188**	12	1.27E-06	5.09E-10
**POM121**	12	1.27E-06	5.09E-10
**RAE1**	12	1.27E-06	5.09E-10
**NUP88**	12	1.27E-06	5.09E-10
**POM121C**	12	1.27E-06	5.09E-10
**NUP210**	12	3.43E-06	4.34E-11
**NUP214**	12	3.43E-06	4.34E-11
**NUP153**	12	3.43E-06	4.34E-11
**NUP62**	12	3.43E-06	4.34E-11
C1D	32	2.88E-09	8.60E-07
MPHOSPH6	33	2.18E-08	8.60E-07
RPS6	34	7.82E-06	1.07E-05
RPS9	35	1.30E-05	1.38E-05
RPS7	35	1.30E-05	1.38E-05
RPS2	35	1.30E-05	1.38E-05
RPS14	35	1.30E-05	1.38E-05
UTP18	39	1.43E-05	8.28E-06
RRP36	39	1.43E-05	8.28E-06
NOL11	39	1.43E-05	8.28E-06
UTP15	39	1.43E-05	8.28E-06
NOL6	39	1.43E-05	8.28E-06
WDR75	39	1.43E-05	8.28E-06
RRP7A	39	1.43E-05	8.28E-06

CIRH1A	39	1.43E-05	8.28E-06
FCF1	39	1.43E-05	8.28E-06
HEATR1	39	1.43E-05	8.28E-06
WDR43	39	1.43E-05	8.28E-06
PPP2R5D	39	0.001598654	1.23E-08

*p-value indicates nominal significance

**enrichment indicates enrichment score.

Gene symbols in bold encode for proteins associated with nucleocytoplasmic shuttling.
